# Multicentric phase II trial of gemcitabine plus epirubicin plus paclitaxel as first-line chemotherapy in metastatic breast cancer

**DOI:** 10.1038/sj.bjc.6601518

**Published:** 2004-01-06

**Authors:** F Cappuzzo, F Mazzoni, A Gennari, S Donati, B Salvadori, C Orlandini, G L Cetto, A Molino, E Galligioni, M Mansutti, S Tumolo, A Lucentini, F Valduga, S Bartolini, L Crinò, P F Conte

**Affiliations:** 1 Division of Medical Oncology, Bellaria Hospital, via Altura 3, 40139-Bologna, Italy; 2 Division of Medical Oncology, Santa Chiara Hospital and University, Pisa, Italy; 3 Ospedale Civile Maggiore, Verona, Italy; 4 Radiology and Oncology Department, Santa Chiara Hospital, Trento, Italy; 5 Division of Medical Oncology, Santa Maria della Misericordia Hospital, Udine, Italy; 6 Division of Medical Oncology, Ospedale Civile, Pordenone, Italy

**Keywords:** advanced breast cancer, epirubicin, gemcitabine, paclitaxel, chemotherapy

## Abstract

In this phase II, multicentre trial, patients with metastatic breast cancer (MBC) were treated with a combination of gemcitabine, epirubicin and paclitaxel (GET). The primary objective of this study was to determine the tolerability and activity in terms of complete responce (CR) and overall response rate of the GET combination in this patient population. Patients with no prior treatment for MBC, and at least one bidimensionally measurable lesion received gemcitabine 1000 mg m^−2^ intravenously (i.v.) over 30 min on days 1 and 4, followed by epirubicin i.v. at 90 mg m^−2^ on day 1, and paclitaxel 175 mg m^−2^ over 3 h on day 1, every 21 days, up to eight courses. From May 1999 to June 2000, 48 patients were enrolled from seven Italian institutions. A total of 297 chemotherapy courses were administered with a median of six cycles patient^−1^ (range 1–8). Seven patients (15%) obtained CR and 27 patients (56%) had partial responce, for an overall response rate of 71% (95% CI: 58.3–83.7). After a median follow-up of 23.7 months (range 7.0–34.4), median progression-free survival was 10.5 months (95% CI: 9.2–11.7), and median overall survival 25.9 months. The main haematological toxicity consisted of grade 3 or 4 neutropenia that occurred in 62% of cycles (22% grade 4 and 40% grade 3). The GET combination is active and well tolerated as first-line chemotherapy for MBC.

In the past 30 years, the diagnosis and treatment of breast cancer have advanced considerably. Systemic chemotherapy is one of the main options of treatment for patients with metastatic breast cancer (MBC). In these patients, combination cytotoxic regimens were shown to achieve higher response rates, and longer duration of response and survival compared to single-agent therapy (Hortobagyi, 1997, 2000). However, these regimens could not change the course of the disease. In the past decade, several newer cytotoxic drugs, including gemcitabine, vinorelbine and the taxanes have been developed. Combinations of these newer drugs with older agents provide enhanced activity with a more favourable toxicity profile for the treatment of patients with MBC.

Traditionally anthracycline-based chemotherapy resulted in higher overall response rates compared with nonanthracycline-containing regimens, with improvements in time to progression and overall survival (Fossati *et al*, 1988; A'Hern *et al*, 1993). In MBC, epirubicin-based chemotherapies are as effective as adriamicin-containing regimens in terms of efficacy with reduced toxicity (French Epirubicin Study Group 1988), and higher epirubicin doses (>50 mg m^−2^) correlate with response rate but not with survival (Focan *et al*, 1993; Marschner *et al*, 1994; Bastholt *et al*, 1996; Brufman *et al*, 1997; The French Epirubicin Study Group 2000). When anthracyclines are combined with taxanes in phase II trials evaluating activity and toxicity in MBC, the results have shown that this combination is very active, with overall response rates ranging from 75 to 95%, and with complete response rates as high as 40% (Gianni *et al*, 1995; Gehl *et al*, 1996; Conte *et al*, 1997; Sledge *et al*, 2003). More recently, six phase III randomised trials compared anthracycline–taxane combinations with standard anthracycline–alkylator regimens (Nabholtz *et al*, 1999; Luck *et al*, 2000; Carmichael, 2001; Jassem *et al*, 2001; Biganzoli *et al*, 2002; Mackey *et al*, 2002).

Only one trial demonstrated a survival advantage for the taxane-containing regimens (Jassem *et al*, 2001), but importantly, the complete response rates in all these trials were lower than expected. Based on these data, we have evaluated the possibility of increasing the activity of anthracycline–taxane combinations by adding a third active drug.

Gemcitabine seemed to be a good candidate because of its low myelotoxicity, favourable tolerability profile, new mechanism of action and interesting single-agent activity. The data from phase II trials show that single-agent gemcitabine produces an objective response rate of 25–46% in MBC; moreover, gemcitabine has a mild toxicity profile with a low incidence of myelotoxicity and nonhaematologic toxicity (Carmichael *et al*, 1995; Blackstein *et al*, 1996; Aapro *et al*, 1998). When administered in association with taxanes in pretreated MBC patients, gemcitabine has shown an overall response rate ranging from 41 to 51% (Rothenberg *et al*, 1998; Mavroudis *et al*, 1999). A previous single-institution phase II trial evaluated the activity and tolerability of the gemcitabine–epirubicin–paclitaxel (GET) combination as first-line chemotherapy in 36 MBC patients. In this trial, gemcitabine was given at 1000 mg m^−2^ on days 1 and 4, plus epirubicin 90 mg m^−2^ on day 1 and paclitaxel 175 mg m^−2^ for 3 h infusion on day 1, every 21 days. This study showed that the addition of gemcitabine to the epirubicin–paclitaxel combination is well tolerated with an overall response rate of 92% and a complete response rate of 31% (Conte *et al*, 2001). In order to validate these data in a larger, multicentre trial, we designed this phase II study to evaluate toxicity profile and activity in terms of overall response rate and complete response (CR) rate of the GET combination as first-line chemotherapy in MBC patients.

## PATIENTS AND METHODS

### Eligibility criteria

Patients with histologically confirmed diagnosis of stage IV breast cancer with evidence of progressive disease (PD), and at least one bidimensionally measurable lesion, were enrolled in the trial. Other eligibility criteria included: age between 18 and 70 years; World Health Organization performance status (WHO-PS) <2; adequate bone marrow reserve (white blood cell (WBC) count >4.0 × 10^9^ l^−1^, neutrophils >2.0 × 10^9^ l^−1^, platelets >100 × 10^9^ l^−1^, haemoglobin >100 g l^−1^), adequate function for liver (bilirubin <1.2 mg dl^−1^) and kidneys (creatinine concentration <1.5 g dl^−1^); normal left ventricular ejection fraction by echocardiography or scintigrafy; estimated life expectancy of at least 12 weeks. No prior chemotherapy for metastatic disease was allowed, but adjuvant treatment, without taxanes, was permitted if stopped at least 6 months before study entry. Adjuvant anthracycline-based therapy was allowed if the total cumulative dose was no more than 240 mg m^−2^ in case of doxorubicin, 360 mg m^−2^ in case of epirubicin, and terminated at least 12 months before study entry. Prior endocrine therapy was allowed. No radiation therapy during the preceding 4 weeks was allowed. Exclusion criteria included: central nervous system metastasis, bone metastases as the only site of disease, previous radiation therapy on target lesions, calcium >11 mg dl^−1^, active infection, pregnancy, breast feeding, history of other cancer, use of any investigational agent in the month before enrollment into the study, previous high-dose chemotherapy with autologous stem cell rescue. Written informed consent was obtained from each patient before entering the study. The study was conducted under the approval of the appropriate ethical review boards and the guidelines for good clinical practice. Recommendations of the Declaration of Helsinki for biomedical research involving human subjects were also followed. This study did not include any patient from previous trials.

### Treatment plan

This was a single arm, multicentre, phase II study of a combination of GET as first-line therapy in patients with MBC. Gemcitabine 1000 mg m^−2^ was administered intravenously (i.v.) over 30 min on days 1 and 4. On day 1, gemcitabine was followed by 90 mg m^−2^ of epirubicin given as i.v. bolus and 175 mg m^−2^ of paclitaxel administered via i.v. over 3 h. Chemotherapy was administered in the outpatient setting, and the cycles were repeated every 3 weeks for a maximum of eight courses. All patients were premedicated with dexamethasone, orphenadrine and cimetidine prior to paclitaxel administration.

Dose adjustments for each subsequent cycle were based on the toxicity observed in the previous cycle. Day 1 doses in subsequent cycles for all three drugs were reduced by 25% in case of febrile neutropenia requiring hospitalisation, grade 4 neutropenia lasting more than 7 days or thrombocytopenia with bleeding. Day 4 dose of gemcitabine was reduced by 50% in case of grade 2 neutropenia or grade 2 thrombocytopenia, and was omitted in case of grade 3 neutropenia or grade 3/4 thrombocytopenia. Doses held due to toxicity or missed were not given at a later time. Treatment was stopped earlier in case of PD, patient refusal or unacceptable toxicity.

### Baseline and on-study evaluation

Baseline evaluations included medical history, physical examination with tumour measurements, WHO performance status evaluation, chest X-ray, complete blood cell count (CBC), liver and kidney function tests, ECG and ecocardiography with L-VEF determination. Staging procedures appropriate to define the extent of metastatic disease, which included computed tomography (CT) and/or resonance imaging studies of the chest, abdomen and pelvis, ultrasounds of liver, bone scans and radiographs of suspicious bone segments were performed in all patients. Complete blood cell count was obtained on days 1 and 4 and subsequently weekly. Blood chemistry and physical examination were performed at the start of each cycle. Toxic effects were assessed according to the WHO Toxicity Criteria (Common Toxicity Criteria 1993). Patients were evaluated for response according to the World Heath Organization (WHO) criteria (Miller *et al*, 1981) after every two courses of treatment. Responses were evaluated by the same assessment methods used to determine the disease status at baseline. A CR was defined as the disappearance of all known disease for at least 4 weeks; a partial response (PR) was defined as at least a 50% decrease in the sum of the products of biperpendicular measurements of all measurable lesions. To be assigned a status of CR or PR, changes in tumour measurements were confirmed by repeat assessments no less than 4 weeks after the criteria for response were first met. Stable disease (SD) was less than 25% change in measurable lesions and no new lesions. Any increase in measurable lesions greater than 25% or the appearance of new metastatic sites was defined as disease progression. In the case of bone metastases, the criteria for CR included clear evidence of complete bone recalcification on X-ray, accompanied by either normalisation of a previously abnormal bone scan or attainment of a near normal architecture of the involved bone lesions on X-ray. After the last course of chemotherapy, patients were followed up every 3 months until death, to monitor progression and survival.

### Statistical analysis

Duration of progression-free survival (PFS) and overall survival was calculated from day 1 of the first drug administration to the first evidence of progression or death, by the Kaplan–-Meier method (Fleming, 1982). From a previous experience of epirubicin plus paclitaxel, the CR rate achieved was 18%. To test an increase in CR rate with this combination from 18 to 30%, the sample size required was 82 patients with an alpha error=0.1 and 1-beta error=0.7.

## RESULTS

### Patient characteristics

From May 1999 to June 2000, a total of 48 patients with MBC and measurable disease were enrolled from seven Italian institutions. The accrual was stopped at this point because on the basis of the observed activity, the probability to obtain a CR rate of 30% was very unlikely. The main patient characteristics are reported in Table 1

**Table 1:** Main patient characteristics

**Characteristics**	**No of patients (%)**
*Total eligible*	48
*Age* (years)	
Median	50
Range	40–68
	
*WHO-PS*	
0	41 (85)
1–2	7 (15)
	
*Hormonal receptor status*	
Positive	29 (61)
Negative	14 (29)
Unknown	5 (10)
	
*Dominant metastatic site*	
Viscera	34 (71)
Soft tissue	14 (29)
	
*Bone metastases*	23 (48)
*Number of metastatic sites*	
1	11 (23)
2	23 (48)
3	14 (29)
	
*Previous therapies*	
Adjuvant chemotherapy (All)	24 (50)
Adjuvant anthracyclin	5 (10)
Adjuvant hormonotherapy	18 (38)
Prior hormonotherapy for metastatic disease	5 (10)

. The median age was 50 years (range 40–68) and the median WHO-PS was 0 (range 0–2). Hormonal receptor status was positive in 29 (61%), negative in 14 (29%) and unknown in five (10%) patients. In total, 14 patients (29%) had at least three metastatic sites, and 34 patients (71%) had dominant visceral disease. A tota lof 24 patients (50%) had received prior adjuvant chemotherapy, including anthracyclines in three cases; and 18 patients (38%) had received prior adjuvant hormonal therapy. Five patients (10%) had failed hormonal therapy for metastatic disease.

### Response to therapy

A total of 297 chemotherapy courses were administered with a median of six cycles patient^−1^ (range 1–8). After chemotherapy, seven patients (15%) obtained a CR and 27 patients (56%) had a PR, for an overall response rate of 71% (95% CI: 58.3–83.7). Nine patients (19%) achieved stable disease, while three patients (6%) progressed during chemotherapy. Two patients (4%) could not be evaluated due to refusal to continue the treatment after the first course of therapy (Table 2

**Table 2:** Response rates

	**No of patients (%)**
Complete response	7 (15)
Partial response	27 (56)
Overall response rate	34 (71)
Stable disease	9 (19)
Progressive disease	3 (6)
Not evaluated	2 (4)

). With a median follow-up of 23.7 months (range 7.0–34.4), median PFS was 10.5 months (95% CI: 9.2–11.7), and median overall survival was 25.9 months (Figure 1

**Figure 1:**
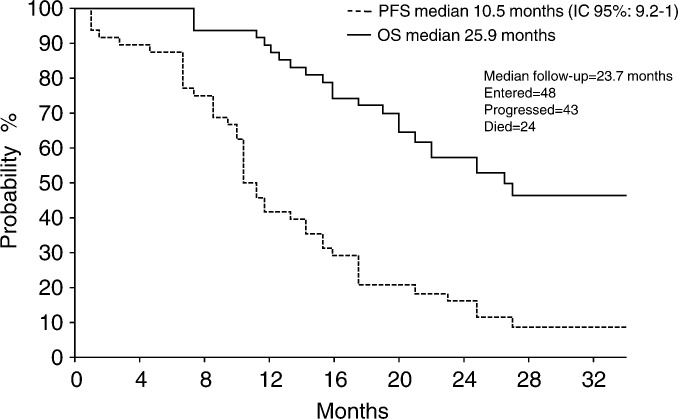
Progression-free survival and overall survival.

).

### Toxicity

Toxicity was monitored in all 297 chemotherapy courses. Table 3

**Table 3:** Haematological and nonhaematological toxicity (% of cycles)

	**Grade 3**	**Grade 4**
Neutropenia	40	22
Anaemia	2	—
Thrombocytopenia	3	1
Nausea/vomiting	2	—
Mucositis	25	6

reports the most significant toxicities encountered with this combination. The main haematological toxicity consisted of grade 3 or 4 neutropenia that occurred in 62% of chemotherapy cycles (22% grade 4 and 40% grade 3). Dose delays or reductions were necessary in 13% of the courses. Three episodes of febrile neutropenia were observed. Other haematological toxicities were uncommon: grade 3–4 anaemia and grade 3–4 thrombocytopenia occurred in 2 and 4% of the courses, respectively.

Nonhaematological toxicity was mostly grade 1 and 2. Grade 2 neurotoxicity occurred in only three patients (6%). Grade 3 and 4 mucositis occurred, respectively, in 12 (25%) and three (6%) patients. Grade 3 nausea/vomiting was observed in one (2%) patient. All patients experienced complete alopecia. One episode of grade 3 cardiac toxicity was observed in a patient pretreated with adjuvant anthracycline; this patient experienced a decline in L-VEF of 25%.

## DISCUSSION

This multicentre phase II trial shows that the addition of gemcitabine to the epirubicin–paclitaxel combination is well tolerated as first-line chemotherapy in MBC patients and feasible on an outpatient basis. Previous phase II trials of the anthracycline–paclitaxel combination in MBC have reported overall response rates of 83–94%, including CR rates up to 41% (Gianni *et al*, 1995; Gehl *et al*, 1996; Conte *et al*, 1997; Sledge *et al*, 1997). More recently, randomised phase III trials comparing anthracycline–taxane combinations with anthracycline–alkylator regimens showed that anthracycline–taxane regimens significantly increase overall response rate, but CR rates remain lower than 20% (Nabholtz *et al*, 1999; Luck *et al*, 2000; Carmichael, 2001; Jassem *et al*, 2001; Biganzoli *et al*, 2002; Mackey *et al*, 2002). In our earlier phase II monocentre study, the GET combination yielded a 92% response rate, including 31% CR (Conte *et al*, 2001). Based on these data, we designed this multicentre phase II study in order to confirm the feasibility, tolerability and activity of this combination. In our previous experience, median PFS was 21 months and, with a median follow-up of 25 months, median survival was not reached (Conte *et al*, 2001). In the present study, the overall response rate achieved was 71% with 15% CR, PFS was 10.5 months and median survival was 25.9 months. Myelosuppression was the most common adverse event, with grade 3–4 neutropenia occurring in 40 and 22% of the courses, respectively. However, neutropenia was reversible and did not require the support of prophylactic G-CSF. Toxicity data from phase III trials comparing anthracycline–taxane combinations with anthracycline–alkylator regimens also indicated that neutropenia is the main haematologic toxicity, but the addition of gemcitabine did not cause increased toxicities. Most notably the incidence of febrile neutropenia was significantly lower (6%) than that observed with other anthracycline/taxane containing triplets utilised both in metastatic and adjuvant setting. In fact, a phase III trial comparing the docetaxel/doxorubicin/cyclophosphamide (TAC) combination to FAC as first-line chemotherapy in MBC patients showed a high incidence of febrile neutropenia (29%) and infection (5%) in the TAC arm (Mackey *et al*, 2002), and the same combination utilized in an adjuvant setting produced comparable toxicities with 24% of patients experiencing febrile neutropenia (Nabholtz *et al*, 2002).

The activity we have observed in this multicentre phase II trial is lower than that reported in the original, single-institution trial in terms of CR rate, progression-free and overall survival. It is well recognised that trials conducted in a multiinstitutional setting produce inferior results as a consequence of differences in patient selection criteria and management. In fact, a multicentre phase II trial of doxorubicin plus paclitaxel conducted by ECOG showed significantly lower overall and complete response rates (29%) in comparison to the original report with the same drug combination (Sparano *et al*, 1999).

In conclusion, the GET combination is feasible, well tolerated and active; further studies are needed to clarify the role of this combination in early stage breast cancer.
